# Optimizing the Tensile Performance of Repaired CFRP Laminates with Different Patch Parameters Using a Surrogate-Based Model

**DOI:** 10.3390/ma18225099

**Published:** 2025-11-10

**Authors:** Zhenhua Yin, Haoying Wei, Zhenyu Ma, Ruidong Man, Jing Yu, Xiaoqiang Wang, Hui Liu

**Affiliations:** School of Mechatronics Engineering, Henan University of Science and Technology, Luoyang 471003, China; a13592016341@163.com (H.W.); 9906625@haust.edu.cn (Z.M.); manruidong@haust.edu.cn (R.M.); 19936422170@163.com (J.Y.); liuhui@haust.edu.cn (H.L.)

**Keywords:** CFRP laminates, tensile damage, nonlinear lamb wave, patch repair, optimization

## Abstract

In this study, nonlinear Lamb wave-based higher harmonic detection is employed to assess the tensile-induced microdamage in patch-repaired carbon fiber-reinforced polymer (CFRP) structures. With respect to the external repair design optimization model based on proxy technology, the minimum nonlinear coefficients are obtained from the optimal patch design parameters, thereby improving the tensile performance of the repaired structure and capturing the repair effect of the patch. First, the nonlinear Lamb wave propagation behaviors of patch-repaired CFRP laminates are assessed under different tensile displacements, and the accuracy of the finite-element model strategy is confirmed by experimental results. Second, on the basis of the tensile displacement induced under the highest nonlinear response, the effects of the radius, thickness and rotation angle of the patch on the secondary and tertiary nonlinear coefficients of the composite glued repair structure and the tensile damage area of the matrix are discussed. After the effects of individual parameters on the patch repair structure are analyzed, the effect of multiple target parameters on the quadratic relative acoustic nonlinearity coefficient of the patch repair structure is investigated via a Latin hypercube experimental design and the Diffuse Approximation method, and the optimal solutions for the mesh parameters of the patch repair structure are successfully obtained, which provides a reference for the multiparameter optimization of patch repair structures in engineering cases.

## 1. Introduction

Carbon fiber-reinforced polymers (CFRPs) are distinguished by their high specific strengths, high specific modulus, high levels of heat resistance, and good fatigue resistance, making them popular in various sectors, including the aerospace, automotive, maritime, and military industries [1,2,3,4,5]. However, in practical applications, CFRPs are typically used in the form of laminates, which are prone to various types of damage during service. After laminates are damaged, their mechanical properties are likely to be compromised [6]. To ensure that the original strength is restored when localized damage occurs, patch repair techniques are often employed to restore a portion of the load-bearing capacity.

Patch repair techniques are cost-effective and straightforward approaches within the spectrum of repair methodologies. This type of method involves removing damaged material through the creation of a circular hole at the affected area, followed by the adhesion of a patch over this hole. This approach is particularly suitable for structures with minimal thickness and less stringent aerodynamic requirements [7]. However, owing to the applied structural alterations, the repaired component is prone to localized stress becoming concentrated around the hole during continued use, which predisposes it to further damage (and is highly likely to become the initiation site for damage propagation). Therefore, to increase the safety and reliability of a structure after repairing it, conducting regular inspections during its subsequent utilization period is critical [8,9].

Nonlinear ultrasonic Lamb wave detection employs nonlinear characteristics such as the higher harmonics generated by the interactions between ultrasonic waves and microcracks. This method determines the fundamental effects of microcracks on the ultrasonic wave propagation process and is widely used in the field of nondestructive testing [10,11,12]. Soleimanpour et al. [13] investigated the higher harmonics generated by contact acoustic nonlinearity during delamination in composite laminates, with numerical and experimental research establishing a numerical model that effectively predicted higher harmonics because of this contact acoustic nonlinearity. Tie et al. [14] used experimental and numerical methods based on nonlinear ultrasonic Lamb waves to detect damage caused by low-velocity impacts in CFRP laminates, with finite-element models and experimental results revealing that the amplitudes of the harmonics and the relative acoustic nonlinearity parameter (RANP) increased with the impact energy, as did the delamination area. Yin et al. [15] assessed the effectiveness of different-sized patch repairs on CFRPs damaged via low-speed impacts via nonlinear Lamb wave analysis. Upon comparing the RANPs obtained for different patch designs, a circular patch with a radius of 2.5 r provided the optimal repair effect. Although scholars have extensively researched the use of nonlinear Lamb waves for detecting damage in carbon fiber-reinforced composites, research on the optimization of the related parameters for conducting nonlinear ultrasonic testing of CFRP laminates after repairing them is scarce.

In addition, to improve the mechanical performance of damaged structures, researchers have conducted extensive studies on patch optimization. Sun et al. [16] optimized the repair parameters of plain woven CFRP laminates via multiscale surrogate modeling and the response surface method to obtain the optimal combination of patch design parameters, significantly reducing the impact energy and delamination area of the repaired laminate. Tie et al. [17] presented a simulation optimization model for the design of external patches, combining cohesive zone models (CZMs) and continuum damage mechanics (CDM) with finite-element modeling (FEM) to produce a simulation model that was consistent with the experimental results, aiming to increase the impact resistance of CFRP laminates with holes. These findings revealed that optimized patches significantly increased the impact resistance of repaired composite laminates. Nonetheless, research on the tensile properties of laminates after undergoing patch repairs is scarce. Li et al. [18] examined the static tensile stress-displacement relationships of bonded structures with patches of various shapes. By constructing models for differently shaped patch bonded structures and predicting the ultimate failure strength on the basis of three-dimensional progressive damage theory, their outcomes suggested that square patch structures yielded the best repair effects. The experiment results conducted on differently shaped patch structures were in agreement with the numerical predictions. However, their patching technique was limited to single-sided applications. Mishra et al. [19] conducted experimental studies on repairs for edge and central cracks in aluminum plates via CFRP patches, employing bidirectional CFRP patches for isotropic cracked plates. The findings indicated that double-sided CFRP patches were most suitable for repairing aluminum plate cracks, with the repaired plates exhibiting an increase in the maximum peak loads for both edge cracks and central cracks relative to those of the unrepaired plates. Bach et al. [20] explored design optimization methods for repairing metal structure fatigue-induced cracks with fiber-reinforced plastic patches. By employing full factorial experiments and genetic algorithms, their design process reduced the patch volume while decreasing the stress intensity factor below the fatigue threshold. However, full factorial experiments require extensive testing, incurring high costs, and the conventional methods have a limited ability to explore the entire design space, often converging to suboptimal local solutions and reaching premature conclusions.

The objective of this study is to exploit the interaction between Lamb waves and matrix tensile damage to detect unrepaired and patch-repaired CFRP laminates by employing numerical and experimental methods that utilize nonlinear higher harmonics. This study examines the relationships among the amplitudes of higher harmonics, RANPs and tensile damage. Initially, the effects of patches on the RANPs in holed composite plates under different tensile displacements are investigated through experimental and numerical methods. A Latin hypercube design (LHD) and the Diffuse Approximation method are subsequently employed. In this study, a surrogate model is constructed to optimize the patch parameters, thereby identifying the optimal patch parameters.

## 2. Theory of Lamb Waves

**Figure 1:**
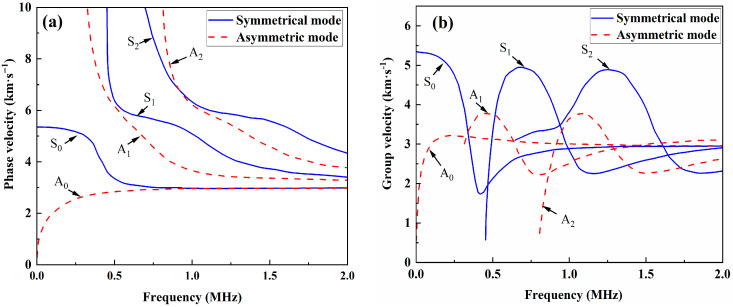
Dispersion curves: (**a**) phase velocity and (**b**) group velocity.

Lamb waves have dispersive and multimodal characteristics during their propagation process in plates, but with the proper selection and precise control of the guided wave frequency and mode, some limitations of the conventional nondestructive testing techniques can be overcome [21,22]. Specifically, the wave speed of a Lamb wave is affected by factors such as the plate thickness, frequency, mode order and material properties. This complex behavior is typically represented by dispersion curves, which graphically show how the phase velocities (or group velocities) of various Lamb wave modes vary with the product of the excitation frequency and the plate thickness. Furthermore, the dispersion curves vary with the properties of the structural material, as well as with the size and shape of the plate. Because they describe the inherent vibrational characteristics of a specific structure, these properties can be used to determine the excitation frequency of the actuator for generating the desired Lamb wave mode. Therefore, a key issue concerning the use of Lamb waves for nondestructive testing is to excite a single Lamb wave mode within regions of minimal dispersion [23,24,25]. In this study, after the material properties of the composite plate are selected, numerical methods are employed to plot the dispersion curves for the composite material, as shown in Figure 1.

As Lamb waves propagate, the greater the number of modes contained in the received signal, the greater the energy dissipation rate. Furthermore, the energy attenuation observed in low-frequency modes is less than that in high-frequency modes, and low-frequency modes have better penetration capabilities [15]. Higher frequencies cause Lamb waves to attenuate more quickly within the sample, making long-distance detection less feasible. As the product of the frequency and thickness increases, the probability of multimode Lamb wave excitation in CFRP laminate increases, indicating that low-frequency transducers should be selected for detecting thick plates. Owing to laboratory limitations, the center frequency of the minimum transducer is 0.5 MHz. Therefore, nonlinear ultrasonic higher-harmonic experiments are conducted within the optimal frequency range of 0.1–0.9 MHz. The results indicate that when a 0.5 MHz excitation frequency is selected and a 1 MHz broadband transducer is used simultaneously, the observed higher-harmonic signals are significantly more pronounced.

The generation of nonlinear higher harmonics depends on the occurrence of sufficient interaction between the wave and defects. Owing to its unique out-of-plane vibration characteristics, the A_0_ mode is inherently sensitive to micropores and microcracks, such as matrix cracking induced by tensile loading within CFRP laminates. The excitation and extraction of the A_0_ mode are relatively purer processes, contributing to increased accuracy and repeatability in nonlinear coefficient measurements. Moreover, when the A_0_ mode is chosen and the fundamental frequency is 0.5 MHz, the corresponding phase velocities of the second harmonic component at 1 MHz and the third harmonic component at 1.5 MHz are very similar, satisfying the principle of phase velocity matching. Hence, considering the transducer frequency and the characteristics of the composite laminate, 0.5 MHz is used as the excitation source frequency in this study.

On the basis of the isotropic assumption of nonlinear acoustics theory, the attenuation that occurs during propagation can be disregarded when the strain is relatively low. Assuming that the guided wave propagates in the positive *x*-direction in a homogeneous medium, by combining the one-dimensional wave equation of ultrasonic guided waves [26] with Hooke’s law, an approximate perturbation solution to the one-dimensional longitudinal nonlinear wave equation for isotropic solids can be derived under one-dimensional conditions [27].

(1) $$\beta^{\prime }=\frac{8A_{2}}{k^{2}A_{1}^{2}x}$$

(2) $$\delta^{\prime }=\frac{48A_{3}}{k^{3}A_{1}^{3}x}$$

where *A*_1_, *A*_2_, and *A*_3_ represent the amplitudes of the fundamental wave, second harmonic, and third harmonic, respectively. *β*′ denotes the second-order absolute acoustic nonlinearity parameter, *δ*′ indicates the third-order absolute acoustical nonlinearity parameter, *x* represents the signal propagation distance, and *k* denotes the wavenumber of the incident acoustic wave, which is defined as follows:

(3) $$k=\omega /c_{p}$$

where *ω* represents the signal frequency and *c_p_* denotes the group velocity of the longitudinal wave.

As shown in Formulas (1) and (2), when the wavenumber *k* and distance *x* are kept constant, to quantitatively express the nonlinear interaction characteristics of Lamb waves and material damage, RANPs are used. This approach determines the extent of waveform distortion when an ultrasonic wave passes through the damaged material and is defined as follows:

(4) $$\beta =\frac{A_{2}}{A_{1}^{2}}$$

(5) $$\delta =\frac{A_{3}}{A_{1}^{3}}$$

where *β* and *δ* represent the second RANP and third RANP, respectively, which are commonly referred to as the second nonlinearity parameter and third nonlinearity parameter, respectively. In this study, when ultrasonic waves propagate in a patch-repaired structure with tensile damage, microcrack damage, and delamination, nonlinear effects can occur, leading to waveform distortion and the generation of higher-order harmonics. Within this context, *β* and *δ* reflect the intensity of these higher-order harmonics. Higher *β* and *δ* values indicate more severe microincompleteness within the material, suggesting a higher density and magnitude of microdamage. Conversely, lower *β* and *δ* values indicate that under these patch design parameters, the material generates less microdamage when subjected to tensile loads. Moreover, dislocation motion is suppressed, internal microcracks are less likely to initiate and propagate, and the final tensile strength of the material is significantly enhanced. Therefore, the RANPs can be used to characterize the extent of tensile damage in patch-repaired structure.

Assuming that the RANP predicted response value of the nonlinear Lamb wave is *x* and the actual value calculated through simulation is *a*; then, the relative error *ε* is as follows:


(6) $$\varepsilon =\frac{\left|x-a\right|}{a}\times 100\%$$

## 3. Finite-Element Simulation

**Figure 2:**
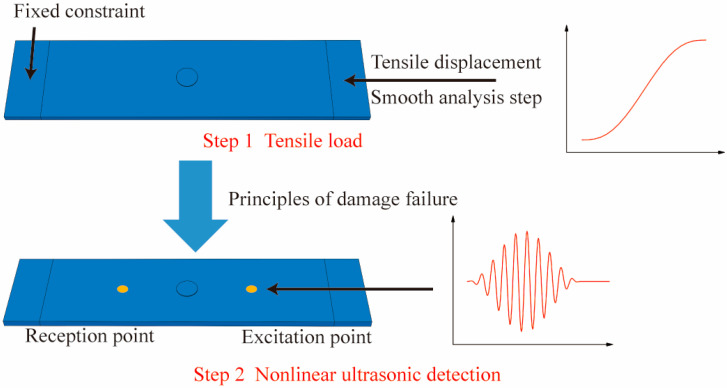
Finite-element modeling process.

In this study, ABAQUS 2021 is employed to conduct a nonlinear Lamb wave-based finite-element simulation of tensile damage in adhesive repair structures. The propagation of Lamb waves is a transient issue that occurs over a very brief period. The use of an explicit solver can yield more accurate results. The finite-element modeling process, as shown in Figure 2, comprises two primary analysis steps: tensile loading and nonlinear ultrasonic detection. This approach enables the use of nonlinear Lamb waves to evaluate the tensile damage suffered by patch-repaired structures under different tensile displacements and to assess the effects of various patch design parameters on the tensile damage in patch-repaired structures.

### 3.1. Finite-Element Model for Tensile Testing

**Figure 3:**
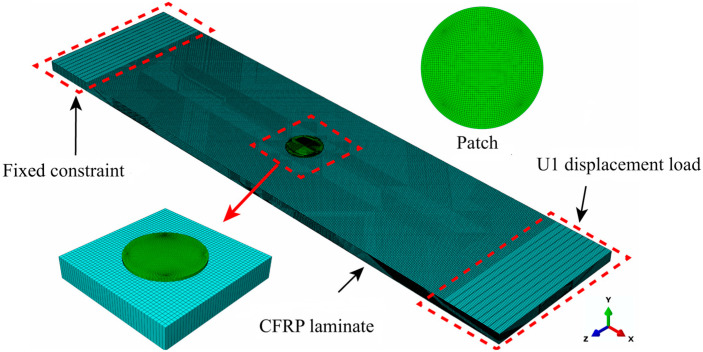
Finite-element model of the composite material-based patch repair structure.

A finite-element model of the composite material-based patch repair structure is shown in Figure 3. The dimensions of a single layer of the laminated plate are 200 mm × 50 mm × 3.6 mm; the plate comprises 24 layers, with a central hole possessing a radius of 3 mm. The patch has a radius of 6 mm with a single-layer thickness of 0.15 mm, totaling 3 layers. The material properties are established for continuous shell elements and adhesive layer parameters, as shown in Table 1. The layup sequence is [45/0/−45/90]_3s_, and the mesh is subdivided into 24 layers along the thickness direction, with the mesh properties assigned to SC8R reduced integration elements.

The Hashin damage failure criterion [28,29] has been widely applied in finite element simulations to predict the initiation and progression of interlaminar damage and ultimate failure in CFRP laminates with different patches. In this study, the adhesive layer is modeled with a cohesive zone model (CZM), and zero-thickness COH3D8 bonded layer cells are chosen to simulate debonding between the patches and parent laminate, as well as the damage between the parent laminate layers. The evolution trend of tensile damage in the matrix is predicted via the failure criterion built into ABAQUS software. The upper and lower clamping surfaces at both ends of the sample are coupled with a reference point, a fixed constraint is applied to the left reference point, and tensile displacement is applied to the right reference point along the length of the sample.

**Figure 4:**
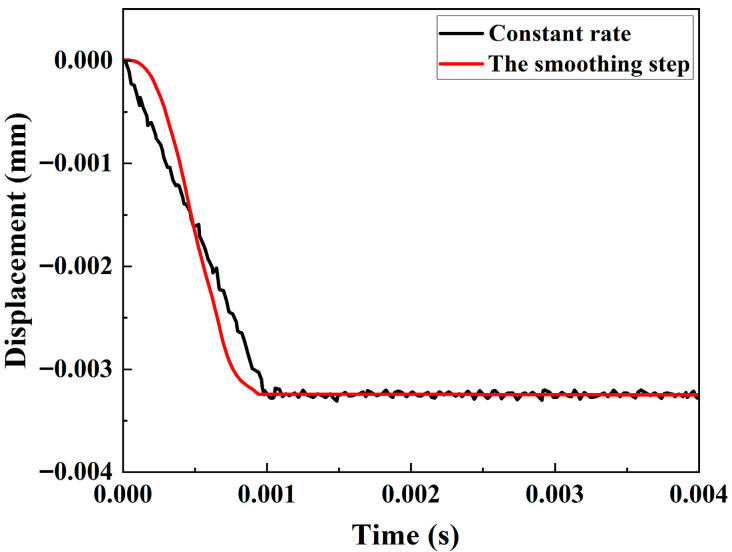
Time-displacement relationship between the receiving points for the two loading modes.

Owing to the cross-sectional constriction of the sample during the tensile process, which generates a Poisson effect, maintaining a constant loading speed for the sample throughout the tensile process results in an instantaneous surge in the acceleration of the plate at the end of the extension stage, leading to minor vibrations on the surface. These vibrations can interfere with the detection signals [30]. Therefore, a smoothing step is performed to eliminate the minor vibrations caused by loading, preventing adverse effects on the subsequent Lamb wave detection step. The smoothing step enables the smoothing of linear data, ensuring that the first and second derivatives of the curve become smooth. As a result, when displacement loading is applied, the sample does not experience a sudden change in its velocity, which would cause intense vibrations. The results of linear loading are compared with those of smoothing step-based loading in Figure 4, which indicates that the smoothing step can effectively eliminate vibrations at the surface reception points of the sample.

### 3.2. Nonlinear Lamb Wave Detection

In accordance with the tensile process, a detection analysis step, where Lamb waves are excited at the excitation point on the right side of the sample for inspection purposes, is performed. The Lamb waves are excited in an antisymmetric mode, which is achieved by applying a displacement perpendicular to the upper surface of the sample at the designated node. The equation for the Lamb wave excitation signal is as follows:

(7) $$u\left(x,t\right)=0.5A\left[1-\cos\left(2\pi ft/N\right)\cdot \sin\left(2\pi ft\right)\right]$$

where *A* represents the modulation amplitude, *f* indicates the fundamental excitation frequency, and *N* denotes the number of cycles. In this study, a 10-cycle, 0.5 MHz Hanning window-modulated signal is selected as the excitation signal. After it is emitted from the excitation point, the signal propagates through the region with the hole and the patch, eventually reaching the receiving point. The distance between the excitation point and the receiving point is 60 mm. During this process, the Lamb waves interact nonlinearly with any damage that is present. The Lamb wave signals at the receiving point are subjected to data processing and analysis [31].

The size and quality of the mesh directly affect the accuracy and stability of the nonlinear propagation results. Therefore, the mesh size of the CFRP laminates is divided according to the mesh size requirements of the Lamb wave propagation characteristics. The model must satisfy the following requirements: the cell mesh size and time step size must be sufficiently small with at least 10 element nodes per wavelength. To obtain the harmonics generated by contact sound waves, owing to the presence of nonlinear effects, the dimensions of each element of the mesh in the finite-element model should satisfy the following condition:

(8) $$\lambda_{\min}\geq 10\max(L_{x},L_{y},L_{z})$$

where *λ_min_* represents the minimum wavelength and *L_x_*, *L_y_* and *L_z_* represent the distances between two adjacent nodes in the *x*, *y*, and *z* directions, respectively. Another essential condition is that the time step must satisfy the following criterion:

(9) $$\lambda_{\min}=\frac{c_{p}}{f_{\max}}$$

(10) $$\Delta t\leq \frac{\min(L_{x},L_{y},L_{z})}{c_{p}}$$

where *c_p_* represents the group velocity of the Lamb wave.

According to Equations (8)–(10), to achieve increased computational efficiency, the element size in the nonlinear Lamb wave detection region where the patch is located is 0.5 mm (as shown in Figure 3), whereas the element size in the remaining regions is 2 mm. The mesh size of the patch is 0.5 mm, and the time step for the nonlinear Lamb wave detection process is 10^−8^ s.

**Figure 5:**
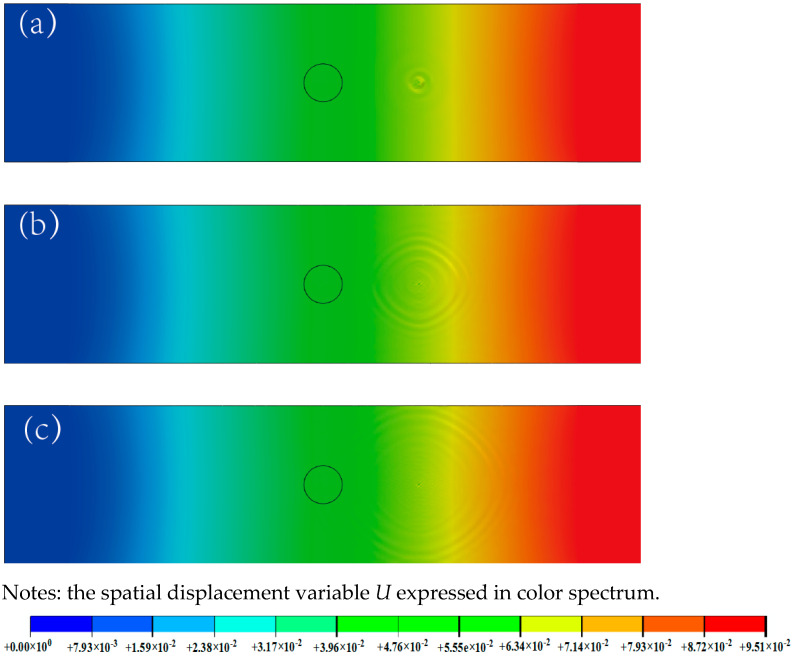
Propagation of Lamb waves in a bonded repair sample: (**a**) 10 μs, (**b**) 20 μs, and (**c**) 30 μs.

The propagation of Lamb waves on the surface of a tensile-repaired bonded sample is shown in Figure 5. The black circles indicates the position of the circular patch. The harmonic amplitude of frequency mixing configuuration superimpositions at differrent Lamb wave propagation in CFRP are presennted using rainbow spectrum of displacement variable *U*. The blue cells indicate no harmonic amplitude and no damage; red cells indicate high mixing harmonic amplitude and significant damage. The nephograms produced at 10 μs, 20 μs, and 30 μs during the propagation process are selected. As the Lamb wave propagates gradually from right to left, it passes through the bonded repair area and interacts with the tensile damage in the matrix. This interaction induces higher harmonic components in the Lamb wave signal, resulting in an increase in the amplitude of the higher harmonics in the received signal spectrum. By performing a frequency-domain analysis of the signals received at the reception points, the nonlinear coefficient can be calculated to evaluate the tensile damage in the composite material sample.

## 4. Experimental Work

### 4.1. Specimen Preparation

**Figure 6:**
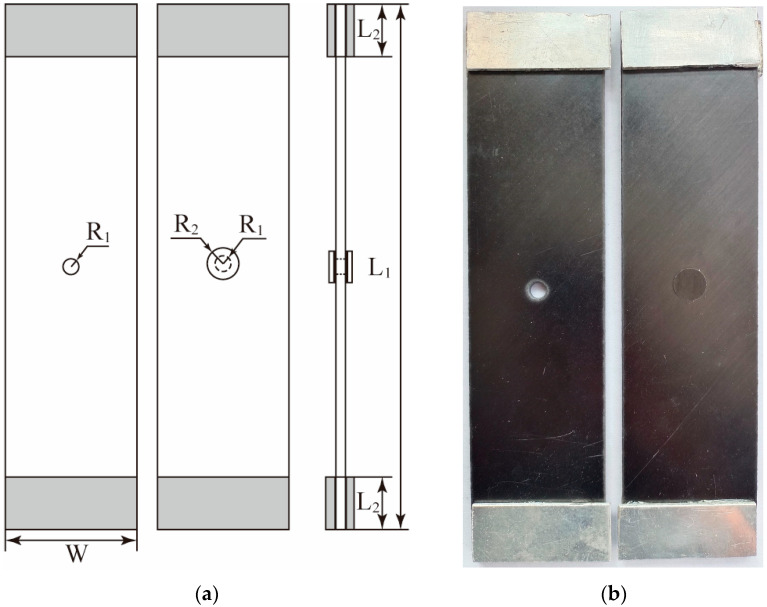
Schematic diagram: (**a**) schematic diagram and (**b**) physical diagram.

The parent laminate, patch and reinforcing piece of the composite patch-repaired structure sample are cut by a high-pressure water jet cutting machine (Anhui Aoyu CNC Technology Co., Ltd., Chuzhou, China). The parent laminate is a model T300/7901 CFRP, the layup sequence is [45/0/−45/90]_3s_, the thickness of a single layer is 0.15 mm, the total thickness is 3.6 mm, and the material properties are listed in Table 1. The material of the patch is the same as that of the parent laminate, the layup sequence is [0/90/0], the thickness of a single layer is 0.15 mm, the total thickness is 0.45 mm, the radius is 6 mm, and the reinforcing piece is an aluminum alloy plate. The tensile specimen of the composite glued repair structure is designed according to the ASTM-D3039 standard [32] test method for determining the tensile properties of polymer matrix composites and the sizes of the transducers, as shown in Figure 6. The unrepaired structure is shown in Figure 6a, and the double-sided patch-repaired structure is shown in Figure 6b. The length L_1_ of the sample is 250 mm, the width W is 50 mm, the thickness D is 3.6 mm, and the length L_2_ is 20 mm. The width of the reinforcing piece is the same as that of the parent laminate, and adhesive film is pasted on the front and back sides of both ends to prevent damage to the parent laminate during the clamping process. A damaged hole is located in the center of the parent laminate, its the radius R_1_ is 3 mm, the front and back sides of the hole are each pasted with a layer of patch, and the center of the mesh coincides with the center of the damaged hole. The radius R_2_, which is equal to 2R_1_, is 6 mm, and 5 groups of samples are created. Three parallel samples are prepared for each set of parameters.

### 4.2. Experimental Design

**Figure 7:**
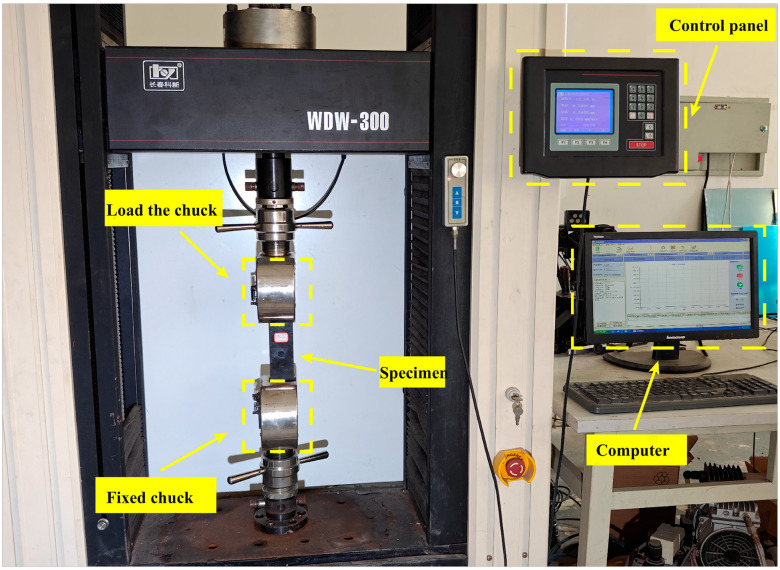
WDW-300 universal testing machine.

A WDW-300 universal tensile machine (Changchun Kexin Testing Instrument Co., Ltd., Changchun, China) is used to carry out tensile experiments on the samples. The experimental machine is shown in Figure 7. The upper and lower chucks are contained in the lower space, the lower chucks are fixed, and the upper chucks can be moved. During the experiment, the test pieces are tightened with the chucks, the lower space is stretched through the computer setting mode, the upper chucks are stretched upward at a speed of 1 mm/min, and the force-displacement curve produced during the loading process is recorded simultaneously. One group of samples with a tensile displacement of 0 is not subjected to an external force load, and the remaining samples are subjected to tensile loads with different displacement lengths by the WDW-300 universal testing machine, resulting in corresponding degrees of damage.

**Figure 8:**
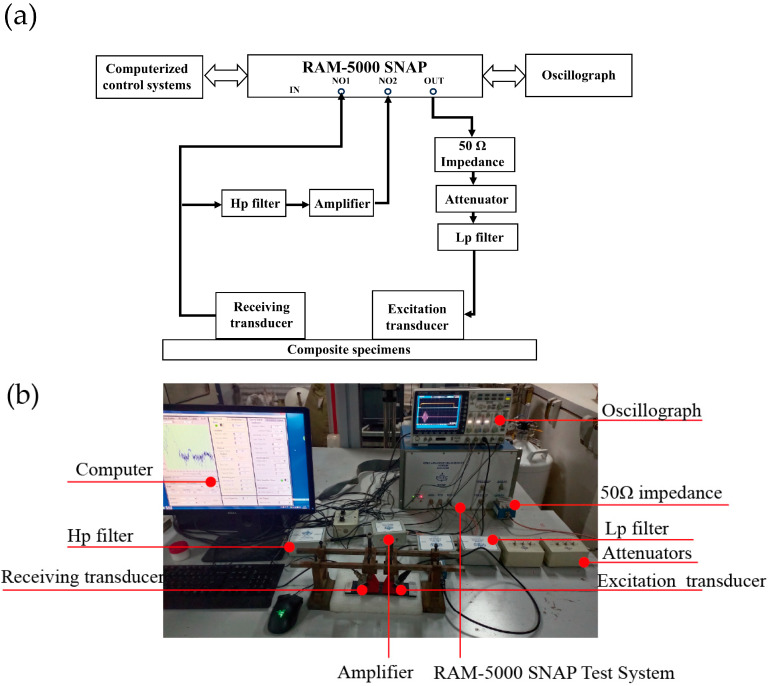
Experimental system for performing nonlinear Lamb wave detection: (**a**) schematic diagram and (**b**) physical diagram.

After tensile tests are conducted on the samples, Lamb wave detection is performed. The experimental setup of the nonlinear ultrasonic Lamb wave detection system is shown in Figure 8. The RAM-5000 SNAP (RITEC Inc., Warwick, RI, USA) nonlinear ultrasonic system can excite the electrical signals of single-frequency sinusoidal longitudinal waves. The computer system can adjust the period and frequency of the signals. To minimize the harmonic generation, the excitation signal displayed on the oscilloscope should not overlap with the reception signal produced during propagation.

In this study, a 10-cycle Hanning window-modulated signal is used as the excitation signal. The signal passes through a 50 Ω impedance device and then through an attenuator and a 0.5 MHz low-pass filter group, to remove the high-frequency interference generated by the device. Among these, the 50 Ω standardized impedance is used for matching to maximize the power transmission level and minimize the signal reflections along the transmission path. The low-pass filter group eliminates the high-frequency harmonic interference signals and errors generated by the RITEC detection system itself, and its effect is particularly significant, increasing the accuracy of the experimental detection results. The signal is then excited via a 0.5 MHz excitation transducer (Olympus NDT, A414S, RITEC Inc., Warwick, RI, USA), with 7501 high-vacuum silicone grease applied as a coupling agent between the transducer and the sample. A specifically designed fixture is used to ensure complete coupling between the transducer and the sample. The signal propagates from the excitation point through the region of the hole and the patch to a 1 MHz broadband reception transducer (Olympus NDT, A407S, RITEC Inc., Warwick, RI, USA), with a distance of 60 mm between the excitation point and the reception point. The excitation transducer, reception transducer, and area to be tested are aligned in a straight line. The signal is received by the receiving transducer after passing through the area to be tested on the sample, processed through a 1 MHz high-pass filter and preamplified by 30 dB, to block and weaken the low-frequency interference signal. The received signal is then extracted and processed by the RAM-5000 SNAP nonlinear ultrasonic detection system, with the results shown on an oscilloscope. The frequency spectrum is shown on the computer control panel.

Before testing, an identical measurement system is run on undamaged samples to maximize the suppression of harmonic noise and experimental system-generated harmonics. This ensures that the nonlinear coefficient enhancement observed in the subsequent damaged samples is indeed primarily attributable to material damage, enabling the reliable extraction of genuine higher-order harmonic information characterizing material damage.

## 5. Results and Discussion

First, on the basis of the experimental and numerical results, the nonlinear Lamb wave propagation behavior observed in CFRP laminates repaired with patches under different tensile displacements is assessed. The accuracy of the finite-element modeling strategy is confirmed by comparing the results. With the highest nonlinear response tensile displacement, this study investigates the degrees of tensile damage suffered by perforated composite plates with different patch design parameters via RANPs, ultimately determining the optimal patch design parameters.

### 5.1. Validity of the Finite-Element Model

As shown in Table 2, the peak tensile load obtained from both finite-element simulation and experiment increases with the tensile displacement, leading to a greater degree of tensile damage. A comparison between the data obtained from the finite-element simulation and the experimental data reveals that the maximum relative error in the peak load is only 3.60%, which is less than 5%. This error primarily originates from the nonuniformity of the composite material prepared in the experiments, as well as the external environmental factors generated during tensile testing, which are inevitable. This error range is acceptable in engineering applications, revealing that the simulation model is highly reliable for simulating the tensile mechanical behaviors of composite materials. Additionally, the tensile analysis step and the boundary conditions imposed on the integrated simulation model are considered reasonable. These findings further prove that the FE simulation strategy used to study the tensile behavior of CFRP laminates repaired with different patches is reliable. In the following study, under the same tensile displacement conditions, i.e., a tensile displacement of 1 mm, a nonlinear ultrasonic high-order harmonic detection analysis is performed on tensile samples with different patch design parameters.

**Figure 9:**
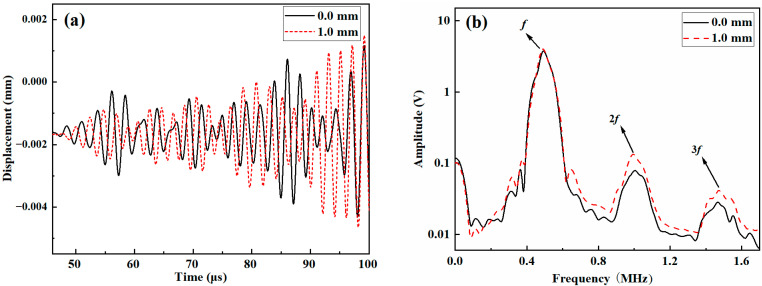
Comparison between the signals received for samples with and without tensile damage: (**a**) time-domain waveforms and (**b**) frequency-domain waveforms.

Nonlinear Lamb wave detection is subsequently conducted on samples with no tensile displacement and those with a tensile displacement of 1 mm. The time-domain waveforms derived from the experiments and simulations are shown in Figure 9a. The waveforms indicate that no significant indicators differentiate the signals acquired from the stretched and unstretched samples. To analyze the differences in frequency components, the time-domain waveforms are converted to spectra via the Fast Fourier Transform (FFT). The frequency-domain waveforms derived from the experiments for the samples with no tensile displacement and those stretched to a displacement of 1 mm are shown in Figure 9b. In this figure, the fundamental frequency amplitude, the second harmonic, and third harmonic amplitudes can be clearly identified, facilitating the analysis of the acoustic nonlinearity coefficient.

### 5.2. Analysis of the Tensile Results

**Figure 10:**
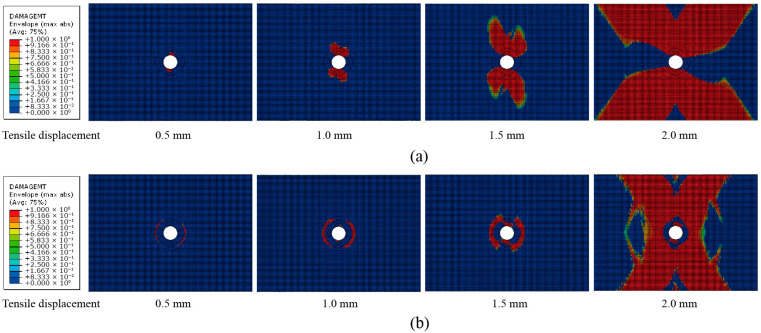
Damage contour plots of the matrix under different tensile displacements: (**a**) unrepaired structure and (**b**) adhesive repaired structure.

**Figure 11:**
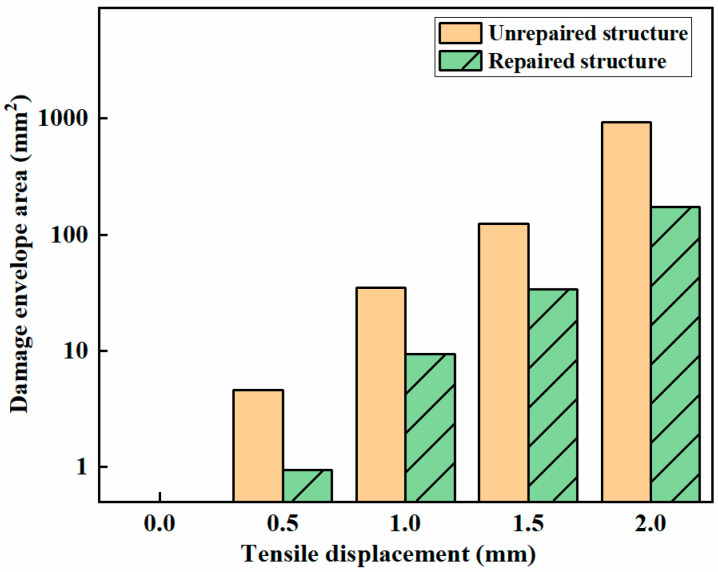
Matrix damage areas obtained under different tensile displacements.

Nonlinear Lamb wave detection is performed on both unrepaired and repaired samples subjected to tensile displacements of 0.5 mm, 1 mm, 1.5 mm, and 2 mm. The simulated damage contour plots of the matrix under different tensile displacements for the unrepaired and repaired samples are shown in Figure 10. A comparison between these plots reveals that the damage area in the unrepaired structure initially appears at the top and bottom edges of the hole. In contrast, for the patched structure, the damage area initially appears around the patch. As the tensile displacement increases, the damage area gradually expands from around the hole, eventually extending to the top and bottom edges. Additionally, the damage areas in the unrepaired samples are larger than those in the adhesively repaired samples. Figure 11 shows the envelope areas of the matrix damage under different tensile displacements, indicating that the adhesive repair technique effectively reduces the microdamage caused by tensile loads and can increase the tensile resistance of composite materials with holes.

**Figure 12:**
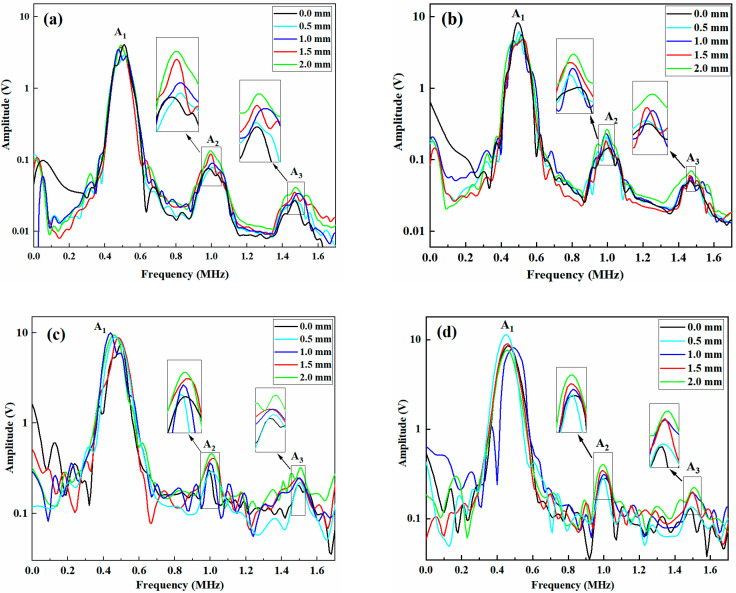
Spectra of the samples under different tensile displacements: (**a**) unrepaired sample (simulation), (**b**) unrepaired sample (experiment), (**c**) repaired sample (simulation), and (**d**) repaired sample (experiment).

The spectra of the detection results obtained for the unrepaired and repaired samples, both simulated and experimental, are shown in Figure 12. Under identical conditions, the amplitude of the second harmonic is consistently greater than that of the third harmonic, and the amplitude tends to increase with increasing tensile displacement. The reason is due to tensile loading on the sample induces microcracks in the damaged areas. As the tensile displacement increases, the crack area gradually increases, resulting in a continuous increase in the amplitudes of the second and third harmonics [33].

**Figure 13:**
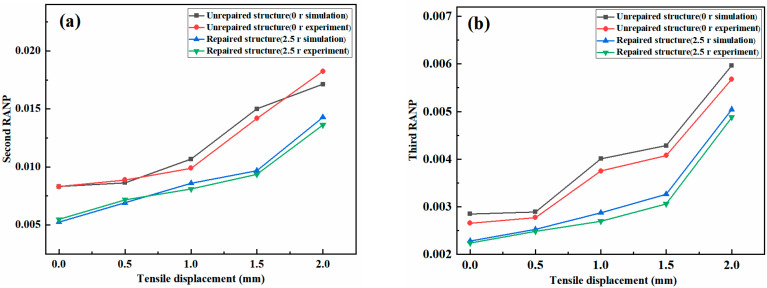
Comparison of nonlinear coefficients between the simulation and the experiment: (**a**) second nonlinear coefficient and (**b**) third nonlinear coefficient.

Figure 13 shows a comparison of the second and third nonlinear coefficients in unrepaired and repaired samples under different tensile displacements obtained from simulations and experiments clearly reveals that the experimental results exhibit the same trend as the simulation results. Both display an increase in tensile damage as tensile displacement increases, accompanied by a corresponding rise in the nonlinear coefficient. This indicates that the increase in tensile damage with increasing tensile displacement is accompanied by a corresponding increase in the nonlinear coefficient. This finding indicates that the nonlinear coefficient can reflect the degree of tensile damage sustained by the material. For intact plates subjected to tensile displacement loading, the primary damage mechanism is matrix tensile damage. Prior to macroscopic fracture occurrence, the tensile displacement, damage level, and the nonlinear coefficient are positively correlated.

The second RANP and third RANP data derived from the experiment and simulation presented in Figure 13 are extracted, as shown in Table 3. The results of the analysis indicate that the maximum error between the experimental and simulation results obtained for the second RANP is 6.30%, and the maximum error induced for the third RANP is 6.69%. Owing to the influences of nonlinear Lamb wave detection environmental factors in the experiment, compared with the RANPs obtained in the numerical simulation, the relative error is acceptable within a certain range, indicating that the integrated finite-element model more accurately simulates the experimental results. These findings further demonstrate the notion that finite element simulation methods can be used to effectively establish models for detecting tensile damage and acoustic nonlinearity in patch-repaired samples.

### 5.3. Effects of the Patch Parameters on MRANPs

#### 5.3.1. Effect of the Patch Radius on the Nonlinear Coefficients

**Figure 14:**
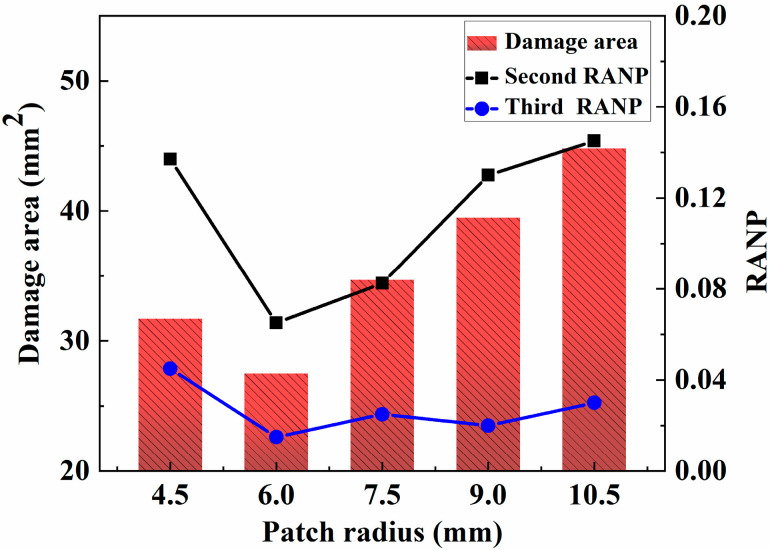
Effect of the patch radius on the glue repair structure.

The results of the previous analysis reveal that the second RANP and third RANP tend to increase but then decrease with increasing tensile displacement. To make the final effect more obvious, a tensile displacement of 1 mm, which corresponds to the highest value of the nonlinear coefficient, is selected for follow-up experiments; the size of the master plate is also consistent with the model, size, etc., employed in the previous experiment, and the center hole R is 3 mm. Typically, the patch radius is a multiple of the radius of the damaged hole. To explore the effect of the patch radius on the tensile performance of the matrix of the adhesive repair structure, the patch rotation angle is maintained at [0], the patch thickness is 0.45 mm, and the other parameters remain unchanged. The complementary radii are 4.5 mm, 6.0 mm, 7.5 mm, 9 mm and 10.5 mm for finite-element modeling, and the relationships between the tensile damage area of the matrix, the quadratic and tertiary nonlinear coefficients and the patch radius are analyzed. A comparison between the tensile damage area of the matrix and the secondary and tertiary nonlinear coefficients of the porous composite board calculated via simulations with different filling radiiis shown in Figure 14.

As shown in Figure 14, with increasing patch radius, the damage area and the quadratic nonlinear coefficient tend to decrease, followed by a subsequent increase. When the radius of the patch increases from 1.5 R to 2.0 R, as the radius of the filler increases, the tensile forces around more holes increase, and the stress concentration at the edge of the hole weakens. However, a critical value exists for the size of the filler radius. If the patch radius exceeds this value, as the bonding area between the patch and the laminated plate with a hole increases, the laminated plate with a hole resists tensile displacement because of the patch bonding induced during the tensile process, and the area in which the shear force is generated increases. Thus, the patch repair becomes meaningless. These findings also explain why the damaged area and the nonlinear coefficient increase as the radius increases from 2.0 R to 3.5 R. Therefore, when the radius of the patch is 6 mm, the damaged area and the nonlinear effect of the Lamb wave reach the minimum values, and the repair effect increases.

#### 5.3.2. Effects of the Sheet Thickness on the Nonlinear Coefficient

**Figure 15:**
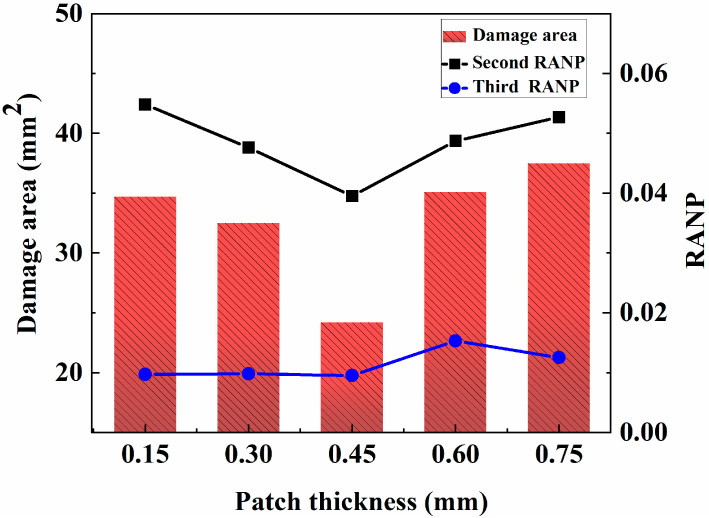
Effect of the patch thickness on the adhesive repair structure.

The strength of the repair structure is affected by the thickness of the repair sheet. The manufacturing process used for composite materials shows that their thickness relies on the thickness of a single layer and the number of layers. In practical applications, the thicknesses of different layers are essentially equal. The thickness of composite plywood is often an integer multiple of the thickness of a single layer. Therefore, to explore the effect of the patch thickness on the tensile performance of the repair structure, the number of layers is increased to control the layer thickness. Because the thickness of a single layer is 0.15 mm, the thicknesses are 0.15 mm, 0.3 mm, 0.45 mm, 0.6 mm and 0.75 mm. As clearly discussed in the previous section, the sample repaired with a radius of 2.0 R has good repair performance. Consequently, the radius of the patch is maintained at 6 mm, the other conditions remain unchanged, and a finite-element simulation analysis of the repair structure is carried out. A comparison between the tensile damage area of the matrix and the secondary and tertiary nonlinear coefficients simulated and calculated for porous composite boards with different thicknesses is shown in Figure 15.

As shown in Figure 15, with increasing patch thickness, the damage area and the quadratic nonlinear coefficient tend to decrease first but then increase. When the patch thickness increases from 0.15 mm to 0.45 mm, its tensile strength increases, which shares the tensile force around more holes and reduces the stress concentration phenomenon at the edge of the hole. However, the patch thickness has a critical value. If the patch thickness exceeds this value, the strength of the path becomes excessive. From 0.45 mm to 0.75 mm, although the stress concentration at the hole edge reduces, the excessive strength causes the absorbed stress to decrease and transform into shear stress between the patch and the surrounding laminated plate, increasing the tensile damage area of the matrix and increasing the weight of the structure. This finding explains why the damage area and the nonlinearity coefficient increase during the process of shifting from 0.45 mm to 0.75 mm. The reason why the increasing trend of the cubic nonlinear coefficient is not obvious later is the same as the previously mentioned explanation. Therefore, when the patch thickness is 0.45 mm, the damage area and the nonlinear effect of the Lamb wave reach the minimum values, and the repair effect increases.

#### 5.3.3. Effects of the Patch Rotation Angle on the Nonlinear Coefficients

**Figure 16:**
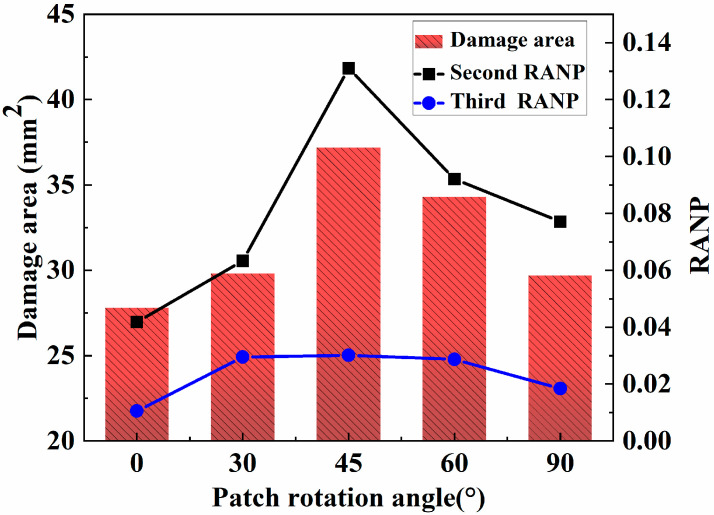
Effect of the patch rotation angle on the glued repair structure.

Owing to the anisotropic nature of composite materials, their mechanical properties can vary significantly in different directions. The tensile strength in the direction parallel to the fiber direction is high, whereas it is low when perpendicular to the fiber orientation. Therefore, the orientation of the repair patch affects the strength of the repair structure. For a circular patch in the layer direction of [0], the rotation angle between the direction of the fiber and the stretching direction of the sample affects the strength of the repair structure. Therefore, to explore the effect of the patch thickness on the tensile properties of the patch repair structure, the rotation angle between the fiber direction of the patch and the tensile direction of the sample is defined as the patch rotation angle. By changing the rotation angle of the patch, patch rotation angles of 0°, 30°, 45°, 60° and 90° are obtained in this study. As shown in the previous section, the samples repaired with 0.45 mm thick patches exhibit good repair performance. Thus, while maintaining a patch radius of 6 mm and a patch thickness of 0.45 mm, and keeping the other conditions unchanged, a finite-element simulation analysis of the patch repair structure is carried out. Figure 16 shows a comparison the tensile damage area of the matrix and the secondary and tertiary nonlinear coefficients of porous composite boards simulated and calculated with different rotation angles.

As shown in Figure 16, as the rotation angle of the supplement increases from 0° to 90°, the damage area and the second RANP and third RANP tend to increase first but then decrease. The reason is that the low tensile damage of the matrix at 0° is attributed to the high tensile strength observed in the direction of the fiber, which shares more of the tensile force around the hole. With increasing rotation angle, the direction of the fibers increasingly deviates from the main load direction, and the load-bearing capacity decreases to its lowest level at approximately 45°. When the rotation angle continues to increase to 90°, although the fiber direction is perpendicular to the main load direction, the repair effect is improved again because of the formation of a cross-layered structure. Thus, the tensile damage suffered by the matrix decreases at this time. A comparison between the repair effects observed at different rotation angles reveals that the repair effect produced at 0° is the best. Therefore, when the rotation angle of the patch is 0°, the damaged area and the nonlinear effect of Lamb waves are minimal values, and the repair effect increases.

## 6. Optimization of the Patch Parameters

### 6.1. Surrogate Model Optimization Based on the Diffuse Approximation Method

Previous studies have revealed that the patch repair technique can reduce the degree of matrix tensile damage observed in perforated composite laminates and result in smaller second and third nonlinear coefficients. Therefore, after the feasibility of the finite-element model is validated, the tensile displacement with the highest nonlinear coefficient is selected in this section. We then conduct a simulation analysis and investigate the effects of three parameters—the patch radius, patch thickness, and patch ply orientation—on the matrix tensile damage and nonlinear coefficients of bonded repair structures. The patch parameters are optimized with a focus on decreasing the nonlinear coefficients and exploring the optimal parameter combination for the patch.

**Figure 17:**
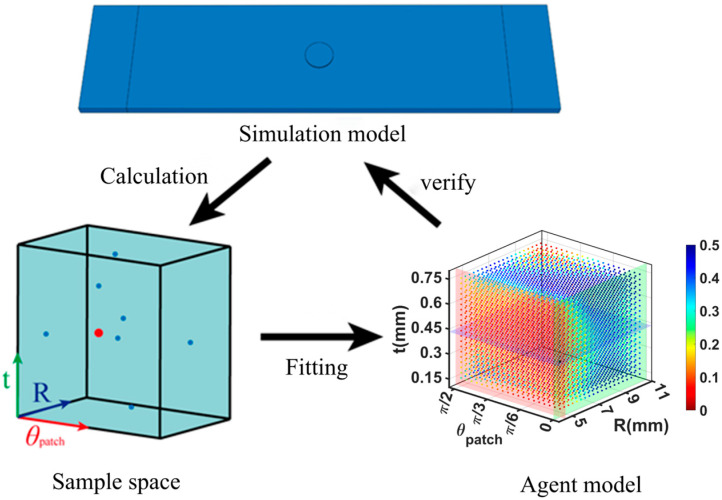
Optimization flowchart.

An optimization scheme based on a Latin hypercube design (LHD) and the Diffuse Approximation method is employed in this study. The flowchart for optimizing the surrogate model is shown in Figure 17. The optimization process is as follows.

1. A series of sample points are selected using the Latin hypercube sampling design.
2. Results are obtained for each sample point through a finite-element simulation.
3. The sample space is constructed using quadratic fitting on the basis of the sample point results.
4. The optimization results are validated.

First, the experimental Latin hypercube design is used to select *n* mesh parameters for analysis purposes, and *m* numerical points are selected within the value range of each parameter to form a vector [*c*_1_, *c*_2,_ …, *c_n_*]. Thus, the basis vector of the model is $C=[c_{1},c_{2},\ldots ,c_{n}]=[c^{1},c^{2},\ldots ,c^{m}]$

After the sample points are determined, the Diffuse Approximation method is used to construct the proxy model [34]. The basic concept of Diffuse Approximation is to replace an interpolation that is valid for an element with a locally weighted least squares fit that is valid only over a variable number of nodes in a small neighborhood of known points. The approximate function is smoothed by continuous weighting functions evaluated at the known points, and the farther these weighting functions are from the known points, the more their values approach 0, preserving the local characteristics of the approximation. Utilizing MATLAB R2021a software, singular value decomposition (SVD) and subsequent iterative matrix calculations are performed to increase the accuracy of the calculation results.

### 6.2. Analysis of the Optimization Results

The selected patch parameters of the adhesive repair structure are the patch radius *R*, the patch thickness *t* and the patch rotation angle *θ*, where the value of *R* ranges from 4.5 mm to 10.5 mm, the value of *t* ranges from 0.15 mm to 0.75 mm, and the value of *θ* ranges from 0° to 90°. As mentioned in the previous section, if the quadratic polynomial model is used, the base vector and coefficient of the model have 10 terms each, so at least 11 sample points are needed to determine the coefficient of the quadratic polynomial.

Through an ABAQUS finite-element simulation, the quadratic nonlinear coefficients corresponding to these 11 groups of bonded repair structures are calculated, and the results are listed in Table 4. To further evaluate the feasibility of the surrogate model, the relative errors between the predicted values and the actual values are calculated. According to the analysis, the relative errors of the second RANP and the third RANP are less than 10%, which proves that the difference between the predicted and the actual values is small, and that the models are more reliable.

Utilizing the coordinates of the sample points and the magnitudes of the quadratic nonlinear coefficients, a polynomial agent model of the glued repair structure can be derived from the MATLAB script with the following expressions:


(11) $$\begin{array}{l} y_{1}=-0.71361+0.21210x_{1}-0.34505x_{2}+(4.59556\times 10^{-3})x_{3}+(2.50703\times 10^{-3})\\ \;\;\;\;\;x_{1}x_{2}-(5.86260\times 10^{-4})x_{1}x_{3}-(1.28528\times 10^{-3})x_{2}x_{3}-0.012295{x_{1}}^{2}\\ \;\;\;\;\;\;+0.43122{x_{2}}^{2}-(1.28428\times 10^{-6}){x_{3}}^{2} \end{array}$$

(12) $$\begin{array}{l} y_{2}=-0.060396+0.07185x_{1}-0.30975x_{2}-(3.06907\times 10^{-3})x_{3}+0.012214\\ \;\;\;\;\;x_{1}x_{2}-(8.35369\times 10^{-5})x_{1}x_{3}+(2.45836\times 10^{-3})x_{2}x_{3}-(5.07522\times 10^{-3}){x_{1}}^{2}\\ \;\;\;\;\;\;+0.056408{x_{2}}^{2}+(2.24792\times 10^{-5}){x_{3}}^{2} \end{array}$$

In Equations (11) and (12), *y*_1_ represents the quadratic nonlinear coefficient, *y*_2_ denotes the cubic nonlinear coefficient, *x*_1_ indicates the patch radius, *x*_2_ represents the patch thickness and *x*_3_ denotes the patch rotation angle.

The coefficient of determination *R*^2^ (as shown in Equation (13)) is used to evaluate the fitting accuracy of the surrogate model:

(13) $$R^{2}=1-\frac{\sum_{i=1}^{N_{i}}{\left(y_{i}-{\tilde{y}}_{i}\right)}^{2}}{\sum_{i=1}^{N_{i}}{\left(y_{i}-{\bar{y}}_{i}\right)}^{2}}$$

where *N_i_* represents the number of sample points, *y_i_* represents the true value, ${\bar{y}}_{i}$ represents the average value, and *ỹ_i_* represents the response value predicted by the surrogate model. *R*^2^ ∈ [0, 1], with the general requirement that it is greater than 0.9. The closer *R*^2^ is to 1, the more the predicted values approach the true values obtained from the numerical simulations, indicating that the surrogate model has a smaller error and higher accuracy. The *R*^2^ values calculated for the two surrogate models are 0.9830 and 0.9925, indicating that both surrogate models have good fitting performance.

**Figure 18:**
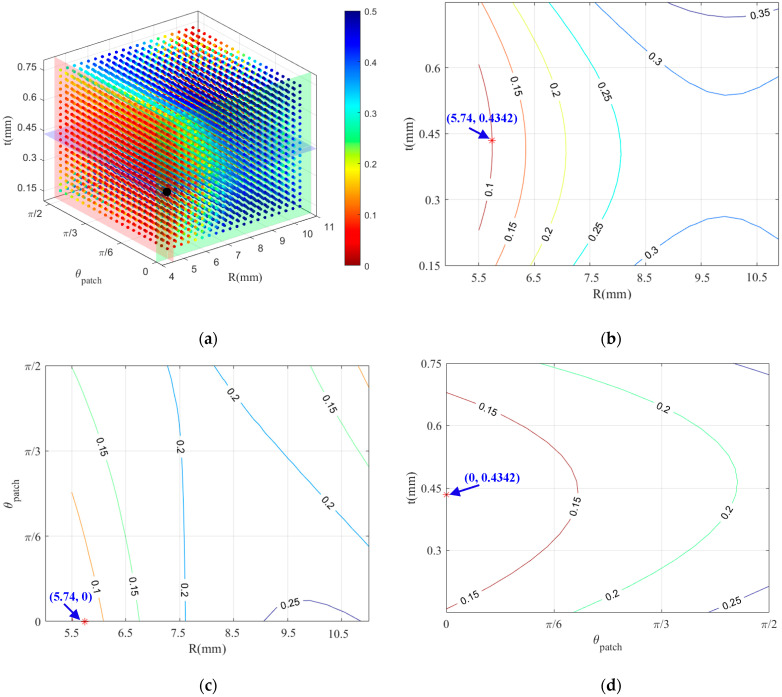
Surrogate model of RANPs (denoted by the color scale) constructed using Diffuse Approximation (**a**) in the (*R*, *t*, *θ*) space, and the projected contours of the RANPs on various parameter planes: (**b**), (*R*, *t*), (**c**), (*R*, *θ*) and (**d**), (*t*, *θ*).

A quadratic polynomial can be used to fit the values of the nonlinear coefficients for all the points in the entire space. To present the entire surrogate model, three-dimensional plots are created via MATLAB software, with various shades representing the magnitudes of the values. The multi-objective optimization model should simultaneously satisfy the optimal values of second RANP and third RANP. As shown in Figure 18a, a darker red color indicates lower nonlinear coefficients, whereas a darker blue color indicates higher nonlinear coefficients. By searching for the minimum value across the entire three-dimensional space, the black point corresponds to the sample point with the minimum nonlinear coefficients, which represents the optimal solution.

In the surrogate model that targets the nonlinear coefficients, the points within the three orthogonal planes that contain the optimal solution are extracted to create contour plots of the patch radius R, patch thickness T, and patch rotation angle *θ* on the RANPs are constructed, as shown in Figure 18b–d. Red stars in these contours (corre sponding to the black dot in Figure 18a) denote the optimal solution identified by minimizing the surrogate model. In these contour plots, the red asterisk indicates the optimal solution. The contour lines are sparser and a more gradual change in the gradient, indicating independent variable has no significant impact on the nonlinear ultrasound coefficients. As shown in Figure 18b, the contour gradient change vary more dramatically in the R direction than in the T direction, highlighting that R exerts a stronger influence on the RANPs with steep curves. Therefore, the effect of the patch radius on the nonlinear coefficient is greater than that of the patch thickness. The contour gradient decreases from the lower left corner to the other directions, with a sharp decreases toward the lower left and upper right corners (Figure 18c). A local minimum appears in the upper right corner, but the overall minimum is located in the lower left corner. The gradient change and the steepness of 2D contour plots in the R direction are significantly greater than those in the *θ* direction, indicating that the influence of R has a greater weight than that of *θ*. The overall influence of the patch radius on the nonlinear coefficient is slightly greater than that of the patch rotation angle. As shown in Figure 18d, the contour gradient change in *θ* is significantly greater than that in the T direction, indicating that the effect of patch rotation angle on the nonlinear coefficients are greater than that of the patch thickness. The magnitude of influence on the RANPs is in the following order: R > *θ* >T. The final optimal solution obtained for the nonlinear coefficients includes a patch radius of 5.74 mm, a patch thickness of 0.4342 mm, and a patch rotation angle of 0°.

**Figure 19:**
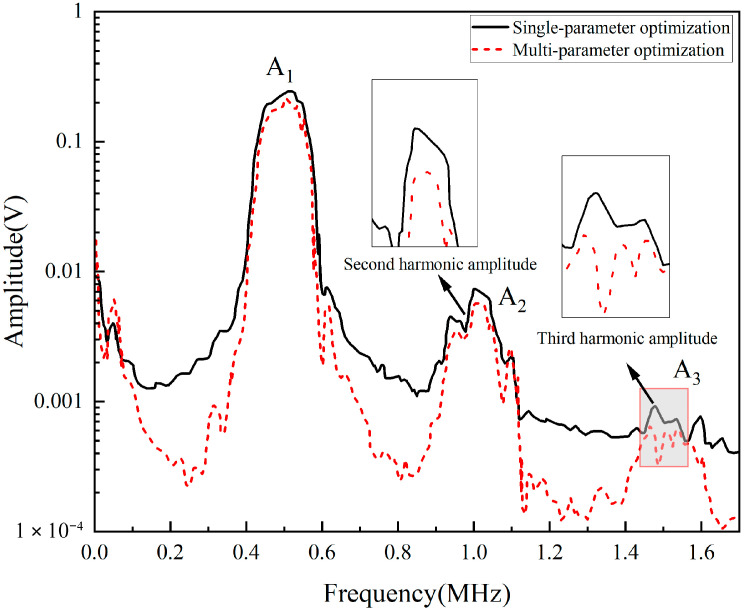
Comparison between the spectra produced during single-parameter and multiparameter optimization.

The optimal solution is substituted into the bonded repair structure model for finite-element simulation analysis, and the results are compared with the previously obtained single-parameter analysis results. The resulting frequency spectrum comparison is shown in Figure 19. Under the same baseline, the amplitudes of the second and third harmonics are decrease after optimization scheme is implemented. The corresponding amplitudes are extracted from Figure 19 and substituted into Equations (4) and (5) to calculate the quadratic and cubic nonlinear coefficients, with the results presented in Table 5. The comparison reveals that the quadratic nonlinear coefficient of the patch-bonded repair structure after performing multiparameter optimization is reduced by 26.74% compared with that obtained after performing single-parameter optimization, whereas the cubic nonlinear coefficient is reduced by 35.01%. Therefore, when the surrogate model is used, the optimal patch parameters can reduce the quadratic nonlinear coefficient, decreasing the tensile damage area of the matrix under the same displacement and achieving the best repair effect.

## 7. Conclusions

In this study, the experimental and simulation results obtained for repaired and unrepaired composite laminated plates under different levels of tensile strain are analyzed, revealing that the relationship between the second and third RANPs and tensile damage increases but then decreases, with general consistency observed between the experimental and the simulation results. After the finite-element model is validated, the design of experiments method, combined with Diffuse Approximation, is used to optimize the patch parameters that target the second and third RANPs, and the results of the two optimization objectives are analyzed. The conclusions are as follows: the second and third RANPs of the patched samples are lower than those of the unrepaired samples, and both RANPs tend to increase with increasing tensile displacement. The results calculated by the established finite element model have an insignificant error compared with the experimental results, indicating the reliability of the simulation process. With the second and third RANPs as optimization targets, the results produced based on the surrogate model show that the optimization result obtained for the second RANP is lower than that obtained for the third RANP. For a T300/7901 composite plate with a thickness of 3.6 mm and a damage hole radius of 3 mm, the bonded repair structure achieves optimal repair performance when the patch radius is 5.74 mm, the patch thickness is 0.4362 mm, and the patch rotation angle is 0°. Compared with the optimal results of the single-factor simulation, the reductions are 26.74% and 35.01%, respectively. The optimal patch design combination for patch-repaired laminates after performing optimization via Diffuse Approximation can significantly reduce the RANPs, which can effectively inhibit the crack damage extension ranges of CFRP laminates under tensile loading, improve the overall mechanical properties and increase the service lives of composite structures. In future research, computer vision-based models, such as the DeepLab and EfficientNet methods [35,36], should be used to automate the crack initiation and growth quantification processes based on high-resolution tensile test images, enabling pixel-level mapping of the microdamage progression to validate and complement nonlinear Lamb wave coefficients.

## Figures and Tables

**Table 1:** Material properties of the T300/7901 CFRP laminates and LJM-170 adhesive films.

T300/7901 CFRP Laminates		LJM-170 Adhesive Films	
Young’s modulus, *E*_11_/GPa	125.90	Tensile stiffness, *K*_nn_/(N·mm^−3^)	10^5^
Young’s modulus, *E*_22_ = *E*_33_/GPa	11.30	Shear stiffness, *K_ss_ = K_tt_*/(N·mm^−3^)	10^5^
Shear modulus, *G*_12_ = *G*_13_/GPa	5.43	Tensile strength, $t_{n}^{max}$/MPa	85
Shear modulus, *G*_23_/GPa	3.98	Shear strength, $t_{s}^{max}=t_{t}^{max}$/MPa	146
Poisson’s ratio, *ν*_12_ = *ν*_13_	0.30	Toughness in tension, $G_{c}^{I}$/(kJ·m^−2^)	0.52
Poisson’s ratio, *ν*_23_	0.42	Toughness in shear, $G_{c}^{II}=G_{c}^{III}$/(kJ·m^−2^)	1.02
Shear strength, *S*/MPa	120		
Density, *ρ*/(kg·m^−3^)	1800		

**Table 2:** Comparison among the tensile test results.

Tensile Displacement/mm	Experimental Load/kN	Simulated Load/kN	Relative Error/%
0.5	28.31	27.29	3.60
1	55.23	54.36	1.58
1.5	80.68	79.69	1.23
2	99.74	97.53	2.22

**Table 3:** Comparison of second RANP and third RANP results under different tensile displacements.

Tensile Displacement/mm	Second RANP			Third RANP		
	Experiment	Simulation	*β*_Second RANP_ (%)	Experiment	Simulation	*δ*_Third RANP_ (%)
0	0.00548	0.00525	4.20	0.00223	0.00228	2.24
0.5	0.00717	0.00692	3.49	0.00248	0.00252	1.61
1	0.00809	0.00860	6.30	0.00269	0.00287	6.69
1.5	0.00935	0.00967	3.42	0.00306	0.00326	6.54
2	0.01362	0.01429	4.92	0.00488	0.00505	3.48

**Table 4:** Structural parameters of adhesive repairs and their corresponding nonlinear coefficients.

Run	*R*/mm	*t*/mm	*θ*/°	Numerical Value		Response Value		Relative Errors (%)	
				Second RANP	Third RANP	Second RANP	Third RANP	*β*_Second RANP_	*δ*_Third RANP_
1	6.07	0.27	4.86	0.0639	0.1162	0.0665	0.11561	4.10	0.51
2	5.14	0.69	16.17	0.0415	0.00742	0.0388	0.00801	6.46	7.96
3	8.52	0.46	70.35	0.0672	0.02551	0.0673	0.02550	0.15	0.03
4	4.63	0.16	51.67	0.0468	0.02594	0.0457	0.02618	2.29	0.92
5	6.36	0.51	76.86	0.0909	0.04004	0.0910	0.04003	0.04	0.02
6	8.17	0.74	65.38	0.1167	0.03257	0.1142	0.03312	2.14	1.69
7	10.17	0.22	38.31	0.0541	0.01161	0.0573	0.01089	5.91	6.16
8	8.96	0.4	25.68	0.1107	0.04426	0.1088	0.04687	1.72	5.91
9	6.73	0.61	43.07	0.1020	0.02692	0.1091	0.02536	6.96	5.81
10	7.35	0.32	88.08	0.0994	0.04829	0.0998	0.04819	0.36	0.21
11	9.41	0.56	21.83	0.1069	0.03272	0.1123	0.03151	5.05	3.69

**Table 5:** Comparison between the single-parameter and multiparameter optimization results.

Project	*R*/mm	*t*/mm	*θ*/°	Quadratic Nonlinear Coefficient	Cubic Nonlinear Coefficient
Single-parameter optimization	6	0.45	0	0.1477	0.07977
Multi-parameter optimization	5.74	0.4342	0	0.1082	0.05184
Reduction rate (%)	—	—	—	26.74	35.01

## Data Availability

The original contributions presented in this study are included in the article. Further inquiries can be directed to the corresponding authors.

## References

[1] Takamura M., Isozaki M., Takeda S., Koyanagi J. (2025). Numerical Analysis on Optimal Adhesive Thickness in CFRP Single-Lap Joints Considering Material Properties. Materials.

[2] Šofer M., Cienciala J., Fusek M., Pavlíček P., Moravec R. (2021). Damage analysis of composite CFRP tubes using acoustic emission monitoring and pattern recognition approach. Materials.

[3] Butenegro J.A., Bahrami M., Abenojar J., Martínez M.Á. (2021). Recent progress in carbon fiber reinforced polymers recycling: A review of recycling methods and reuse of carbon fibers. Materials.

[4] Wang H., Huo S., Chevali V., Hall W., Offringa A., Song P., Wang H. (2025). Carbon Fiber Reinforced Thermoplastics: From Materials to Manufacturing and Applications. Adv. Mater..

[5] Qi J., Li C., Tie Y., Zheng Y., Duan Y. (2021). Energy absorption characteristics of origami-inspired honeycomb sandwich structures under low-velocity impact loading. Mater. Des..

[6] Cui Z., Qi J., Tie Y., Zou T., Duan Y. (2023). Research on the energy absorption properties of origami-based honeycombs. Thin-Walled Struct..

[7] Caliskan U., Ekici R., Yildiz Bayazit A., Apalak M.K. (2021). Numerical model for composite patch repair of notched aluminum plates under impact loading. Proc. Inst. Mech. Eng. Part L J. Mater. Des. Appl..

[8] Hall Z., Liu J., Brooks R.A., Crocker J., Joesbury A., Harper L., Blackman B., Kinloch A., Dear J. (2022). The effectiveness of patch repairs to restore the impact properties of carbon-fibre reinforced-plastic composites. Eng. Fract. Mech..

[9] Pitanga M.Y., Cioffi M.O.H., Voorwald H.J., Wang C.H. (2021). Reducing repair dimension with variable scarf angles. Int. J. Adhes. Adhes..

[10] Nandyala A.R., Darpe A.K., Singh S.P. (2022). Damage severity assessment in composite structures using multi-frequency lamb waves. Struct. Health Monit..

[11] Allen J.C.P., Ng C.T. (2022). Debonding detection at adhesive joints using nonlinear Lamb waves mixing. NDT E Int..

[12] Sampath S., Sohn H. (2023). Non-contact microcrack detection via nonlinear Lamb wave mixing and laser line arrays. Int. J. Mech. Sci..

[13] Soleimanpour R., Soleimani S.M. (2022). Scattering analysis of linear and nonlinear symmetric Lamb wave at cracks in plates. Nondestruct. Test. Eval..

[14] Tie Y., Zhang Q., Hou Y., Li C. (2020). Impact damage assessment in orthotropic CFRP laminates using nonlinear Lamb wave: Experimental and numerical investigations. Compos. Struct..

[15] Yin Z., Li C., Tie Y., Duan Y. (2020). Impact damage detection in patch-repaired CFRP laminates using nonlinear lamb waves. Sensors.

[16] Sun Z., Pi X., Tie Y., Li C. (2023). Study on impact resistance and parameter optimization of patch-repaired plain woven composite based on multi-scale analysis. Polym. Compos..

[17] Tie Y., Hou Y., Li C., Meng L., Sapanathan T., Rachik M. (2020). Optimization for maximizing the impact-resistance of patch repaired CFRP laminates using a surrogate-based model. Int. J. Mech. Sci..

[18] Li C., Zhao Q., Yuan J., Hou Y., Tie Y. (2019). Simulation and experiment on the effect of patch shape on adhesive repair of composite structures. J. Compos. Mater..

[19] Mishra K., Lal A., Sutaria B.M. (2023). Experimental investigation of repair of plate with edge and center crack by surface bounded composite patch. Int. J. Steel Struct..

[20] Do B., Lenwari A. (2020). Optimization of fiber-reinforced polymer patches for repairing fatigue cracks in steel plates using a genetic algorithm. J. Compos. Constr..

[21] Hu Y., Zhu Y., Tu X., Lu J., Li F. (2020). Dispersion curve analysis method for Lamb wave mode separation. Struct. Health Monit..

[22] Li J., Han Y. (2020). Dispersion compensation method for lamb waves based on measured wavenumber. Shock Vib..

[23] Silitonga D.J., Declercq N.F., Walaszek H., Vu Q.A., Saidoun A., Samet N., Thabourey J. (2024). A comprehensive study of non-destructive localization of structural features in metal plates using single and multimodal Lamb wave excitations. Acta Acust..

[24] Shan S., Cheng L. (2020). Mode-mixing-induced second harmonic A0 mode Lamb wave for local incipient damage inspection. Smart Mater. Struct..

[25] Zhao G., Jiang M., Li W., Luo Y., Sui Q., Jia L. (2023). Early fatigue damage evaluation based on nonlinear Lamb wave third-harmonic phase velocity matching. Int. J. Fatigue.

[26] Zeng S.Y., Jia L., Zhang S.Z., Li X.B., Wang M. (2022). Second-order perturbation solution and analysis of nonlinear surface waves. Acta Phys. Sin..

[27] Yin Z., Tie Y., Duan Y., Li C. (2021). Optimization of nonlinear Lamb wave detection system parameters in CFRP laminates. Materials.

[28] Hashi Z. (1980). Failure criteria for unidirectional fiber composite. J. Appl. Mech..

[29] Yin Z., Tie Y., Duan Y.C., Li C., Chen D. (2022). Impact damage assessment in patch-repaired CFRP laminates using the nonlinear Lamb wave-mixing technique. Polym. Compos..

[30] Panella F.W., Pirinu A. (2021). Fatigue and damage analysis on aeronautical CFRP elements under tension and bending loads: Two cases of study. Int. J. Fatigue.

[31] Yin S., Xiao H., Xu C., Wang J., Deng M., Kundu T. (2022). Microcrack localization using nonlinear Lamb waves and cross-shaped sensor clusters. Ultrasonics.

[32] (2014). Standard Test Method for Tensile Properties of Polymer Matrix Composite Materials.

[33] Liu N., Cheng S., Fan J., Zhu Y., Yang N., Pan Y. (2025). Simulation and experimentation of nonlinear Rayleigh wave inspection of fatigue surface microcracks. Ultrasonics.

[34] Sass J., Westphal D., Wunderlich R. (2023). Diffuse Approximations for periodically arriving expert opinions in a financial market with Gaussian drift. Stoch. Models.

[35] Song Z., Zou S., Zhou W., Huang Y., Shao L., Yuan J., Gou X., Jin W., Wang Z., Chen X. (2020). Clinically applicable histopathological diagnosis system for gastric cancer detection using deep learning. Nat. Commun..

[36] Kabir H., Wu J., Dahal S., Joo T., Garg N. (2024). Automated estimation of cementitious sorptivity via computer vision. Nat. Commun..

